# In-Situ Imaging of Molten High-Entropy Alloys Using Cold Neutrons

**DOI:** 10.3390/jimaging5020029

**Published:** 2019-02-16

**Authors:** Nicholas Derimow, Louis J. Santodonato, Benjamin E. MacDonald, Bryan Le, Enrique J. Lavernia, Reza Abbaschian

**Affiliations:** 1 Materials Science & Engineering Department, University of California, Riverside, CA 92521, USA; 2 Advanced Research Systems, Inc., Macungie, PA 18062, USA; 3 Materials Science & Engineering Department, University of California, Irvine, CA 92697, USA

**Keywords:** high-entropy alloys, neutron imaging, HEAs, multicomponent alloys, multi-principal element alloys, MPEAs, complex concentrated alloys, CCAs

## Abstract

Real-time neutron imaging was utilized to produce a movie-like series of radiographs for in-situ observation of the remixing of liquid state immiscibility that occurs in equiatomic CoCrCu with the addition of Ni. A previous neutron imaging study demonstrated that liquid state immiscibility can be observed in-situ for the equiatomic CoCrCu alloy. In this follow-up study, equiatomic buttons of CoCrCu were placed alongside small Ni buttons inside an alumina crucible in a high-temperature vacuum furnace. The mass of the Ni buttons was specifically selected such that when melted in the same crucible as the CoCrCu buttons, the overall composition would become equiatomic CoCrCuNi. Neutron imaging was simultaneously carried out to capture 10 radiographs in 20 °C steps from 1000 °C to 1500 °C and back down to 1000 °C. This, in turn, produced a movie-like series of radiographs that allow for the observation of the buttons melting, the transition from immiscible to miscible as Ni is alloyed into the CoCrCu system, and solidification. This novel imaging process showed the phase-separated liquids remixing into a single-phase liquid when Ni dissolves into the melt, which makes this technique crucial for understanding the liquid state behavior of these complex alloy systems. As metals are not transparent to X-ray imaging techniques at this scale, neutron imaging of melting and solidification allows for the observation of liquid state phase changes in real time. Thermodynamic calculations of the isopleth for CoCrCuNi_*x*_ were carried out to compare the observed results to the predictions resulting from the current Thermo-Calc TCHEA3 thermodynamic database. The calculations show a very good agreement with the experimental results, as the calculations indicate that the CoCrCuNi_*x*_ alloy solidifies from a single-phase liquid when x ≥ 0.275, which is close to the nominal concentration of the CoCrCuNi alloy (x = 0.25). The neutron imaging shows that the solidification of CoCrCuNi results from a single-phase liquid. This is evident as no changes in the neutron attenuation were observed during the solidification of the CoCrCuNi alloy.

## 1. Introduction

The discovery of high-entropy alloys (HEAs) in 2004 [1,2,3,4,5,6] has led to an explosion in research into multi-component alloy systems with equiatomic or near-equiatomic ratios. In a recent review, these alloys showed promising mechanical, thermal, electric, and magnetic properties [7]. These alloys are sometimes also referred to as multiprincipal element alloys (MPEAs), complex concentrated alloys (CCAs), or baseless alloys so as not to place as many restrictions on the design of these multicomponent systems. The intent is to explore the nature of the phase formation of these complex alloys in the search for a better understanding of miscibility as well as what increased properties can be obtained from these baseless alloy mixtures.

The equiatomic CoCrCuFeNi has been extensively studied since its discovery by Yeh et al. in 2004 due to it dendritically solidifying into primary face-centered cubic (FCC) dendrites with FCC interdendrites [3]. These studies include magnetization [8], compressive strength [8,9], calculation of phase diagrams (CALPHAD) [10], recrystallization [11], thermodynamic properties [12], cold rolling [11,13], and rapid solidification [14].

Liquid phase separation (LPS) in CoCrCuFeNi has also been observed during supercooling [14,15,16,17], or by varying concentrations of Fe/Ni [18,19,20]. LPS resulting from additions of Mo [9] and Sn [17] has also been investigated.

The components present in CoCrCuFeNi have been shown to phase separate when present in ternary or quaternary combinations. For example, the equiatomic ternary CoCrCu has been shown to phase separate in the liquid [21,22], indicating the existence of a stable miscibility gap. Equiatomic additions of Ni to CoCrCu have been shown to eliminate LPS and lead to typical dendritic solidification in the CoCrCuNi alloy [20]. In contrast, additions of Fe to CoCrCu have led to stable LPS which is characterized by macroscopic globule-like phase separations present in the microstructure [19,20]. This would lead one to believe that the Ni addition in the CoCrCuFeNi alloy is what stabilizes the solution into a single-phase liquid as CoCrCu [20] and CoCrCuFe [19,20] exhibit stable LPS. However, LPS has also been observed in induction-melted CoCrCuFe_*x*_Ni when x = 0.5 [18] but not when x = 0 (CoCrCuNi, arc-melted) [20] or x = 1 (CoCrCuFeNi, arc-melted) [3]. When Ni is varied, in the case of arc-melted CoCrCuFeNi_0.5_, stable LPS is also observed [18]. It is not immediately clear whether the LPS observed in these alloys is a result of processing parameters or from elemental concentration.

In an effort to elucidate the liquid state behavior of high-entropy and related alloys, the present authors utilized the CG-1D Neutron Imaging Instrument at the High-Flux Isotope Reactor (HFIR) at Oak Ridge National Laboratory to image the melt separation process in-situ [23]. The technique utilizes a high-temperature furnace between a neutron beam and a detector, and was successfully implemented for the in-situ imaging of the liquid phase separation that occurs in equiatomic CoCrCu [23] as this compound has been shown to exhibit a stable liquid phase separation into CoCr-rich and Cu-rich liquids [20,21]. The experiment consisted of arc-melted CoCrCu buttons placed in a small alumina crucible inside a high-temperature vacuum furnace, which were then heated to 1500 °C, and slowly cooled back to room temperature with simultaneous neutron imaging being carried out in 25 °C steps. The technique requires that the neutron transmission percentage between the alloying elements is different enough such that a contrast between the phases can be seen during imaging. The experiment demonstrated that while at high temperatures, the melt separation can be observed in-situ as the CoCr-rich and Cu-rich layers separated and stacked similarly to the classic observations of oil and water [23].

The aim of this follow-up investigation was to apply the techniques for in-situ neutron imaging of melting and solidification to the CoCrCu alloy with buttons of Ni placed alongside the CoCrCu buttons in the crucible, so as to image in-situ mixing of the added Ni to the previously immiscible CoCr-rich and Cu-rich liquid phases. The slow cooling rate paired with neutron imaging of the solidification process will help elucidate the equilibrium solidification behavior of this alloy, as well as image the remixing of the immiscible CoCr-rich and Cu-rich liquids. As neutrons can penetrate through metals, this technique provides a novel solution for observing the liquid state behavior of HEAs.

## 2. Experimental

### 2.1. Sample Preparation and Furnace Setup

**Figure 1:**
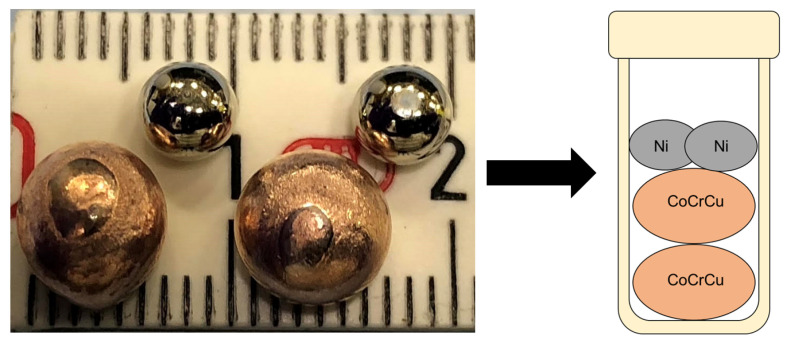
Photograph of the arc-melted CoCrCu buttons next to the remelted Ni buttons prior to the neutron imaging and melting.

Two buttons of CoCrCu were prepared such that their atomic concentrations would remain equiatomic upon the melting of the two Ni buttons added into the crucible to form equiatomic CoCrCuNi (the CoCrCu + Ni buttons were doubled to increase the volume of material inside the crucible). The samples were prepared from elemental purities of Co ≥ 99.9%, Cr ≥ 99.99%, Cu ≥ 99.9%, and Ni ≥ 99.99%. The CoCrCu buttons were arc-melted two times (flipped once) on a water-cooled Cu hearth in a Ti-gettered argon atmosphere. CoCrCu button dimensions were ∼8 mm while the elemental Ni was remelted from chunk form into a smaller spherical shape of ∼5 mm, presented in Figure 1.

**Figure 2:**
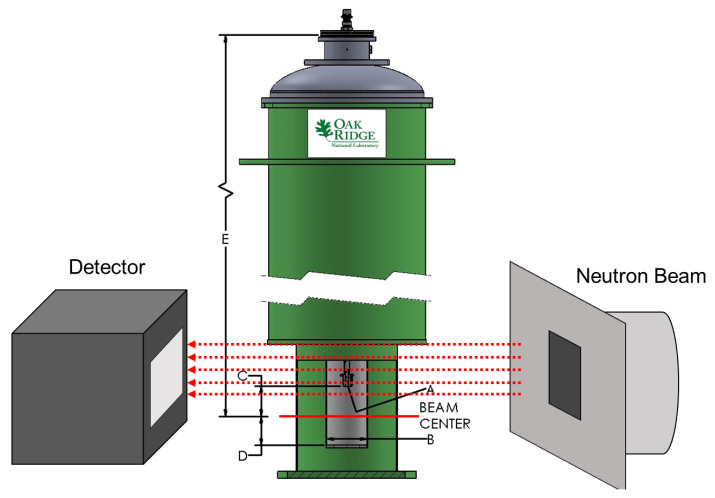
Diagram of the experimental setup at the CG-1D beamline at the High Flux Isotope Reactor (HFIR) at Oak Ridge National Laboratory. The image depicts the high-vacuum Institut Laue-Langevin (ILL) HOT-A furnace placed between the detector and the incident neutron beam slits. (A) Interface connection M8 × 1.25 (male), (B) Bore size diameter = 50 mm, (C) Distance interface to beam center = 31.75 mm, (D) Beam center to sample space bottom = 11.862 cm, E) Distance stick flange to beam center = 41.275 cm. Image of ILL furnace ‘HOT-A’ courtesy of Oak Ridge National Laboratory Sample Environment Group.

**Figure 3:**
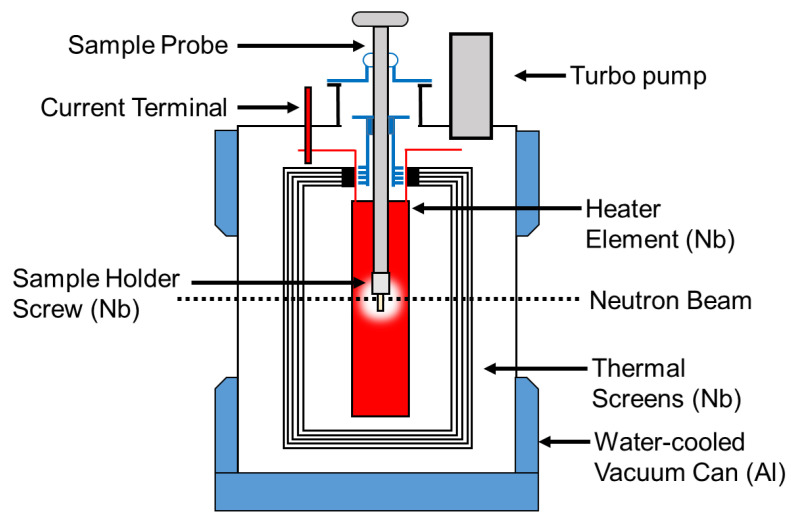
Top loading neutron furnace layout and description of furnace elements. More information pertaining to this furnace can be found in Ref. [24].

The two CoCrCu and two Ni buttons weighing 1.14 g, 1.12 g and 0.38 g, 0.38 g, respectively, were stacked in a small alumina crucible of dimensions 27 mm height, 8 mm inner diameter, and 12 mm outer diameter. The crucible was then closed with a lid of 13 mm in diameter with a height of 3 mm (no vacuum seal). The crucible was then placed inside a top-loading “ILL-type” vacuum furnace customized at Oak Ridge National Laboratory (referred to as the HOT-A furnace), which was then placed between the neutron source and the detector (Figure 2). A more detailed description of the high-temperature furnace is presented in Figure 3. The buttons were heated up to the maximum operating temperature of the furnace (1500 °C) which was continuously pumped during heating to maintain a vacuum of ≈1 × 10^−6^ mbar. Additional experimental details regarding the furnace setup are described in Ref. [24].

### 2.2. Neutron Imaging

The calculations for the transmission of neutrons through the elements/phases in these alloys are presented in Table 1, where Δx is the path length through the attenuating material. The neutron transmission values are calculated from,

(1) $$I\left(\lambda \right)=I_{0}\left(\lambda \right)e^{-\mu (\lambda )\Delta x}$$

where I(λ) and $I_{0}\left(\lambda \right)$ are the transmitted and incident neutron intensities, respectively, for wavelength λ. A more comprehensive description of the attenuation percentage calculation can be found in Ref. [25].

The CG-1D Neutron Imaging Instrument uses a polychromatic neutron beam, which is referred to as “cold” neutrons. The beam passes through a liquid hydrogen cold source which results in a wavelength range of 0.8 < λ < 6 Å, which peaks at 2.6 Å. This wavelength range gives sufficient transmission and contrast for imaging the present elements (Table 1, calculated assuming a peak wavelength of λ = 2.6 Å). Neutron radiographs were acquired using a ^6^LiF/ZnS scintillator viewed by an ANDOR DW936 CCD detector. The detector field of view was a 75 mm square region on the 100 μm thick scintillator screen. The distance from the aperture to the detector, L, was 6.49 m, while the aperture diameter, D, was 11 mm. Therefore, the L/D ratio for this experiment was 599.1. Sample distance to scintillator was approximately 13 cm, resulting in a working spatial resolution on the order of ∼200 μm. The image acquisition was set to acquire 10 radiographs with an acquisition time of 10 s/radiograph (with a 4 second delay between radiographs) every 20 °C from 1000 °C → 1500 °C → 1000 °C with a 5 °C tolerance and ramp rate of 20 °C/min. The acquisition of images in these steps allows for a movie-like series of radiographs to display melting events in-situ [23]. The maximum temperature of 1500 °C was held for an additional 150 radiographs, so there would be sufficient time for mixing before entering the cooling portion of the image acquisition program.

After the heating/cooling cycle, the solidified CoCrCuNi alloy + crucible was removed from the furnace and placed on a rotating stage for additional radiographs such that computed tomography (CT) could be carried out on the solidified alloy. The CT scan was performed at room temperature on the rotating stage from 0 to 360° in steps of 0.20° at a rate of 1 image/step and an exposure time of 20 s per image.

## 3. Results

**Figure 4:**
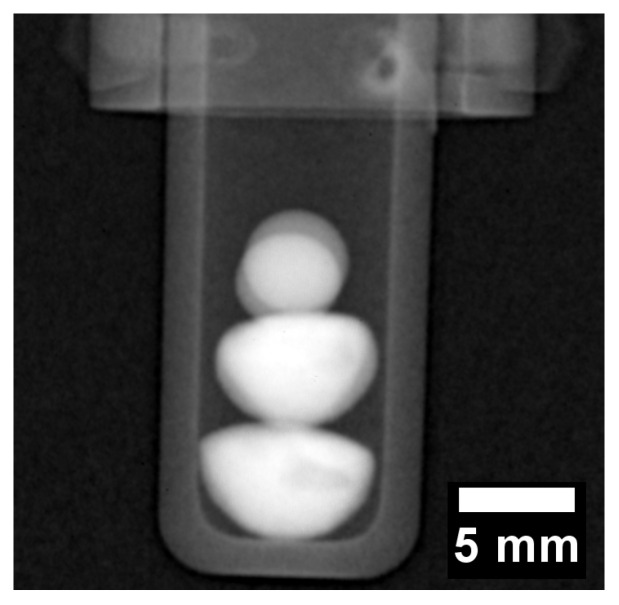
Radiograph of the four buttons stacked inside an alumina crucible such that the heterogeneous CoCrCu are placed on the bottom while the spheres of Ni are placed at the top (the Ni buttons are oriented in the direction of the beam such that they overlap). The darker regions present randomly in the bottom two buttons are the Cu-rich phase that separated in the liquid during arc-melting of the CoCrCu buttons.

The radiograph in Figure 4 displays a negative image of the two CoCrCu buttons stacked with two spherical pieces of Ni placed on top of the two CoCrCu buttons. The two Ni buttons sat in the same path as the neutron beam, therefore there is a slight overlap between the two buttons which causes a slight increase in contrast. The CoCrCu buttons at the bottom of Figure 4 are heterogeneous from the arc-melting process, as indicated by brightness fluctuations in the bottom two buttons in Figure 4. The lighter region pertains to the CoCr-rich solid phase while the darker sides and portions of the inner regions are Cu-rich. This miscibility gap in CoCrCu [21] leads to macroscopic-phase segregation of CoCr-rich and Cu-rich phases during solidification. The differences in phase are distinguishable from the other in Figure 4 due to differences in brightness based on the attenuation of neutrons through each phase in the CoCrCu buttons [23]. The alumina crucible as well as the niobium sample stick are also visible in Figure 4 however do not interfere with the neutron transmission through the buttons.

**Figure 5:**
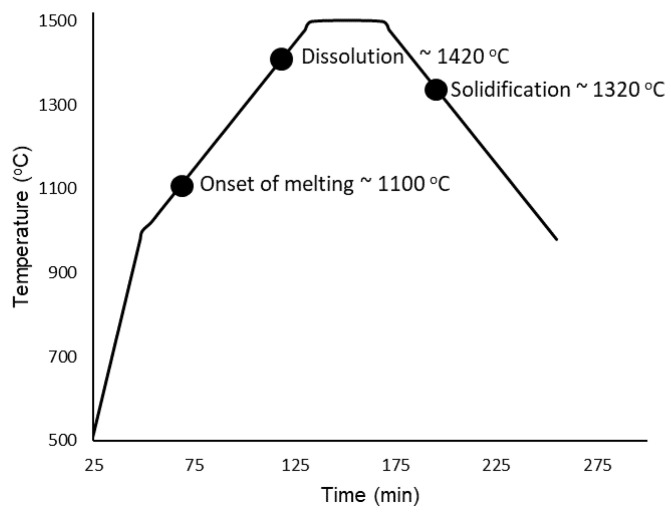
Temperature vs. time of the CoCrCu + Ni samples heated and imaged from 1000 °C to 1500 °C and back down to 1000 °C.

**Figure 6:**
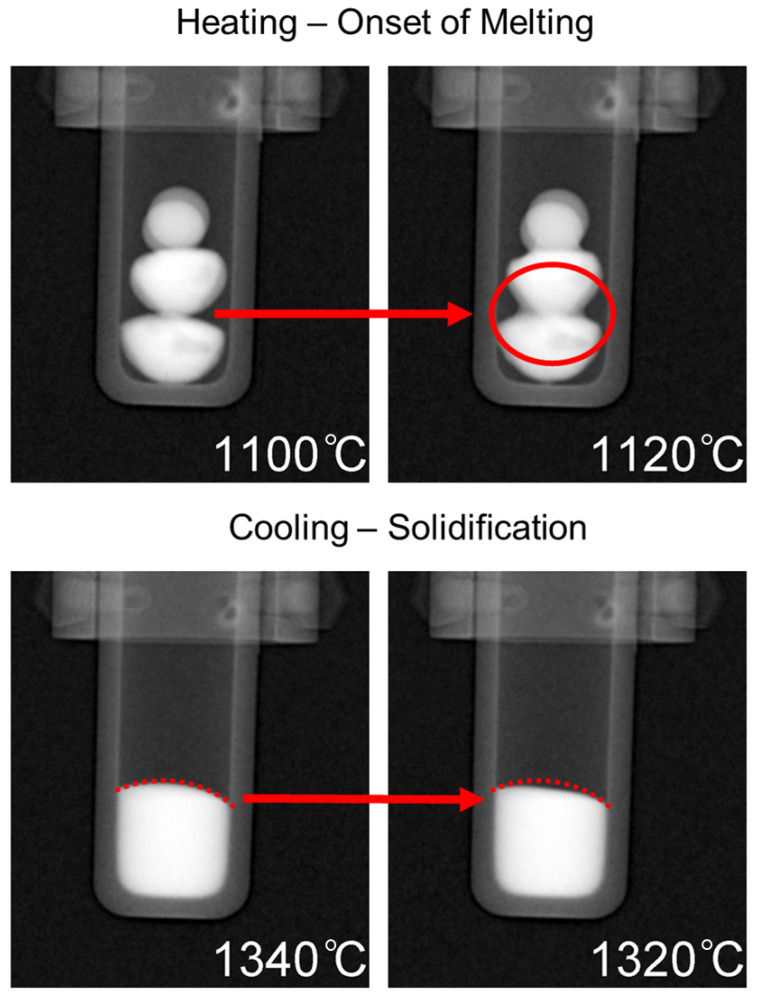
Top: Onset of melting as depicted via the dark phase spreading downwards outlined by a red circle. Bottom: Solidification as indicated by thermal contraction inside the dotted red line.

Heating of the samples began from room temperature up to 1000 °C without any radiographs being taken. Once 1000 °C was reached, the programmed image acquisition process was carried out as described in the experimental details. The heating and cooling cycle is presented in Figure 5 with labels corresponding to the onset of melting at approximately 1100 °C, complete dissolution at ∼1420 °C, followed by solidification at ∼1320 °C. The onset of melting and subsequent thermal contraction due to solidification are indicated by the change in shape of the buttons/melt, and is presented in Figure 6, which is labeled with red arrows to indicate the changes that occurred in the samples during measurement. For the onset of melting, the red arrow in Figure 6 points from the very first slight changes in shape at a 1100 °C radiograph to a radiograph taken at 1120 °C where a red circle outlines the Cu-rich phase on the surface of the CoCrCu buttons that begins to fully melt and spread outwards. As the melting point of pure Cu is 1085 °C, the visual onset of Cu melting at 1100 °C furnace temperature suggests that, during heating, the furnace thermocouple is about 15 °C higher than the sample during heating. If a similar trend holds during cooling, the sample will be approximately 15 °C hotter than the furnace. As such, the accuracy of the temperature measurements is within ±15 °C.

**Figure 7:**
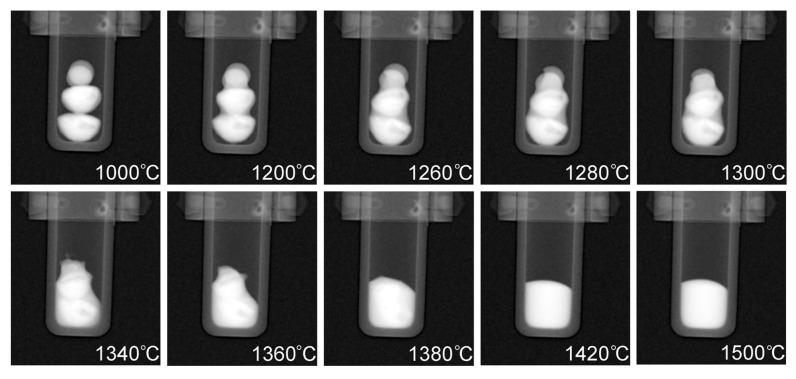
Radiographs taken at increasing temperatures of the CoCrCu + Ni buttons inside the alumina crucible in the HOT-A vacuum furnace. A full movie-like sequence of radiographs can be found in the Supplementary Materials.

Radiographs taken at different temperatures during the heating process are presented in Figure 7. The buttons are in the solid state at 1000 °C, while the subsequent radiographs show different stages of melting as well as the final temperature of 1500 °C which was held for approximately 35 min to ensure sufficient mixing had taken place. Full dissolution of the phases occurred at 1420 °C which is noticeable due to the uniform contrast of the molten pool in the crucible at 1420 °C when compared to the darker (Cu-rich) phases that are present throughout the CoCrCu buttons at the lower temperatures during heating. A full movie-like sequence of the radiographs (recorded in 10 fps → 20 °C steps/s) can be found in the Supplementary Materials.

**Figure 8:**
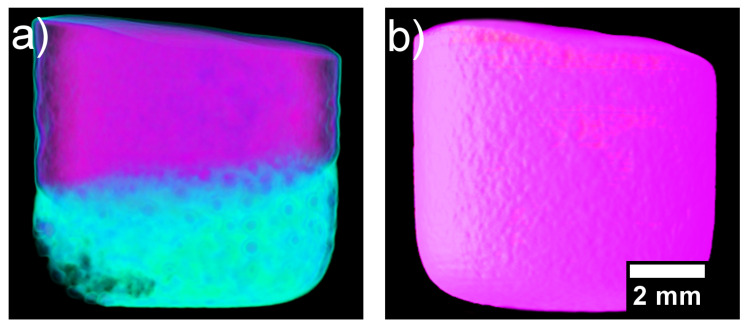
(**a**) Reconstructed computed tomography (CT) of the solidified heterogenous CoCrCu alloy recreated from Ref. [23] with permission from the authors. (**b**) Reconstructed CT of the solidified CoCrCuNi alloy.

After the heating and cooling neutron imaging cycle finished, the samples were taken out of the furnace and radiographed on a 360° rotating stage for CT neutron imaging. The reconstructed CT image of the solidified CoCrCuNi alloy solid is presented in Figure 8. The surface roughness is likely due to thermal contraction paired with the alloy being in contact with the alumina crucible walls. The overall contrast of the sample is uniform throughout as opposed to the initial presence of the different contrast phases (CoCr-rich and Cu-rich) that are present in Figure 4.

## 4. Discussion

The CoCrCuNi alloy has been previously shown to solidify into a uniform dendritic microstructure from arc-melting [20], which is indicative that the microstructure evolved from a single-phase liquid as opposed to the large-scale macroscopic-phase separation that has been observed in similar alloys of CoCrCu [21] and CoCrCuFe [19]. However, there are no reported slow cooling solidification studies on the CoCrCuNi alloy. Previous neutron imaging of the CoCrCu by the present authors resulted in neutron radiographs of the CoCrCu displaying the buttons melting and separating based on density [23]. The stacking of the liquid immiscible phases of CoCr-rich liquid and Cu-rich liquid was characterized by their differences in neutron attenuation. The same experiment was essentially repeated for this work with the equiatomic addition of Ni buttons such that the equiatomic CoCrCu becomes equiatomic CoCrCuNi once melting and dissolution has occurred. This allows for a more equilibrium solidification due to the slow cooling, as well as an opportunity to image the remixing of the immiscible CoCr-rich and Cu-rich liquid phases.

Figure 7 displays how the multiple attenuated phases dissolve into one phase, indicating that the previously immiscible CoCr-rich and Cu-rich phases in the CoCrCu alloy became miscible when equiatomic portions of Ni are added to the system. Although pure Ni has a melting point of 1455 °C, the molten Cu present on the surface of the heterogeneous CoCrCu buttons leads to the dissolving of the Ni buttons, which becomes more visible in Figure 7 at 1260 °C. The CT images were processed using ImageJ software, and are presented in Figure 8. The CT of the alloys was reconstructed from the radiographs of the immiscible solid CoCrCu alloy (recreated from Ref. [23] with permission from the authors), and the solid CoCrCuNi HEA that was radiographed on a rotating stage for this study, which corresponds to the solid phase presented in the bottom right of Figure 6. The crucible was removed from the image after applying color thresholds to reveal the solid CoCrCuNi piece. The CT images represent the final solid forms of the alloys after the in-situ neutron imaging, having been removed from the furnace and imaged while still in the crucible.

Inspection through the cross-sections of the CoCrCuNi revealed no additional phases, indicating that the CoCrCuNi alloy had solidified from a single-phase liquid with no macroscopic-phase separations. This is in contrast to the large phase separation in CoCrCu visible in Figure 8a. For the CoCrCu alloy, the purple coloring indicates the CoCr-rich phase while the green coloring represents the Cu-rich phase. The same look-up tables (LUT) were applied in Figure 8b for the CoCrCuNi alloy, where no macroscopic liquid phase separation can be seen. The results indicate that Ni acts as a solubility pathway for the Cu-rich liquid phase to enter the solution with the overall melt.

**Figure 9:**
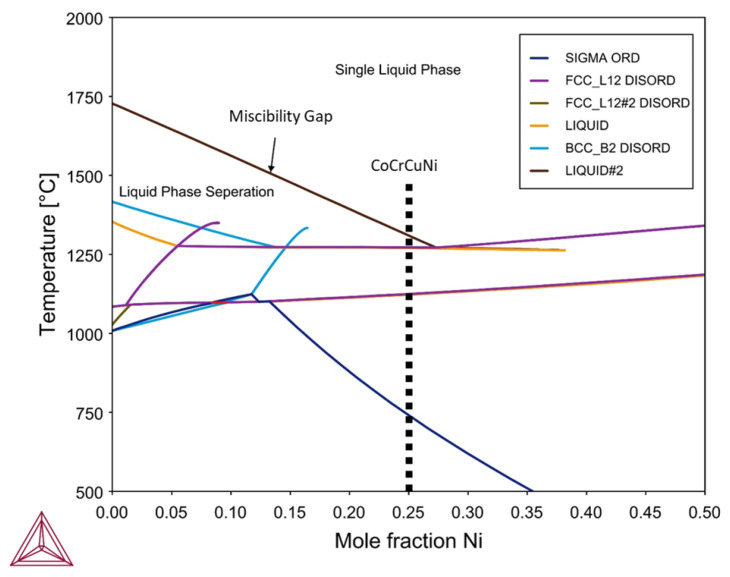
Calculated isopleth of the CoCrCuNi_*x*_ system using Thermo-Calc software. Legend descriptions: SIGMA ORD = ordered σ phase, FCC_L12_ DISORD = disordered FCC phases, BCC_B2_ DISORD = disordered BCC phases.

Thermodynamic calculations for the CoCrCuNi alloy system were performed using Thermo-Calc software using the TCHEA3 thermodynamic database to generate an isopleth of CoCrCuNi_*x*_ such that the left hand side of the diagram (0 at. % Ni) represents equiatomic CoCrCu while the right hand side of the phase diagram would represent pure Ni. The calculated isopleth present in Figure 9 displays the CoCrCuNi_*x*_ system from x = 0 to 0.50 where equiatomic CoCrCuNi corresponds to 25 at. % Ni (0.25 mole fraction) which is indicated by a dotted line in Figure 9. The calculation of the isopleth for this system shows agreement with previous calculations [21] and experiment [23] for the left hand side of the isopleth (CoCrCu), as there is indeed a significant liquid state miscibility gap for the CoCrCu alloy. The calculations also show that there exists a single-phase liquid region above the miscibility gap until approximately 27.5 at. % Ni, which at percentages greater than this would result in a single phase in the molten state with no liquid phase separation at all.

The current experiment reached temperatures of 1500 °C and stayed at this temperature to ensure good mixing between the phases. Slow cooling from 1500 °C to room temperature did not yield any changes in neutron attenuation of the CoCrCuNi liquid, which indicates that there was no observable macroscopic liquid phase separation during solidification of the CoCrCuNi alloy. The CT images presented in Figure 8 compare the equiatomic CoCrCu (Figure 8a) with the equiatomic CoCrCuNi (Figure 8b), and correspond to the left hand side of the isopleth in Figure 9 (0 at. % Ni) and the dotted line representing equiatomic CoCrCuNi (25 at. % Ni), respectively. The CT of CoCrCuNi in Figure 8b shows no macroscopic liquid phase separation, therefore the thermodynamic calculations show good agreement with the experiment, as the miscibility gap line in the calculated phase diagram in Figure 9 ends at 27.5 at. % Ni, and is very close to the experimental concentrations used in this study of 25 at. % Ni.

## 5. Conclusions

The field of high-entropy alloys continues to grow every year, and with this growth comes an enormous amount of never before seen alloy systems with unknown liquid state behavior. As previously shown, neutron imaging can be a very useful technique to elucidate the liquid state behavior of molten alloy systems. With the application of this technique to high-entropy alloys, in-situ imaging of liquid state miscibility has been observed for the first time with the equiatomic additions of Ni to the immiscible CoCrCu system. The current results show the following:

- Neutron imaging can be utilized to image liquid state behavior in high-entropy alloys. Imaging was carried out on equiatomic CoCrCu buttons with equiatomic additions of Ni such that when melting occurred between the alloying elements, full miscibility was achieved.
- In-situ neutron imaging was successfully utilized to image in-situ alloying of Ni into an immiscible CoCrCu system. The synthesis of the CoCrCuNi alloy inside the high-temperature furnace was fully imaged via a movie-like sequence of carefully timed radiographs to display the melting/alloying process.
- Previous neutron imaging experiments of CoCrCu show the liquid phase separation that occurs between CoCr and Cu. The added Ni in this system acts as a solubility pathway for Cu to enter the solution with the rest of the alloying elements.
- In-situ neutron imaging of solidification can provide a novel solution to probe the liquid phases of calculated isopleths and provide valuable insight into the liquid state behavior of HEAs.

## Figures and Tables

**Table 1:** Table of neutron transmission through the CoCr, Cu, Ni, and CoCrCuNi phases.

Composition	Density (g/cm^3^)	Δx (mm)	Transmission
CoCr	8.01	8	10%
Cu	8.96	8	40%
Ni	8.91	5	34%
CoCrCuNi	8.47	8	16%

## Supplementary Materials

The following are available online at http://www.mdpi.com/2313-433X/5/2/29/s1, Video: CoCrCu+Ni.
